# Asia-wide phylogeography of wild boar (*Sus scrofa*) based on mitochondrial DNA and Y-chromosome: Revising the migration routes of wild boar in Asia

**DOI:** 10.1371/journal.pone.0238049

**Published:** 2020-08-24

**Authors:** Sung Kyoung Choi, Kyung Seok Kim, Maryana Ranyuk, Elmar Babaev, Inna Voloshina, Damdingiin Bayarlkhagva, Jong-Ryol Chong, Naotaka Ishiguro, Li Yu, Mi-Sook Min, Hang Lee, Nickolay Markov

**Affiliations:** 1 Conservation Genome Resource Bank for Korean Wildlife, College of Veterinary Medicine, Seoul National University, Seoul, Republic of Korea; 2 National Forensic Service Seoul Institute, Seoul, Republic of Korea; 3 Department of Natural Resource Ecology and Management, Iowa State University, Ames, Iowa, United States of America; 4 Institute of Plant and Animal Ecology Ural Branch of Russian Academy of Sciences, Yekaterinburg, Russian Federation; 5 Caspian Institute of biological Resources of Dagestan Scientific Center of Russian Academy of Sciences, Makhachkala, Russian Federation; 6 Lazovsky State Nature Reserve, Lazo, Primorsky Krai, Russian Federation; 7 Department of the Biology, National University of Mongolia, Ulaanbaatar, Mongolia; 8 Wildlife Research Centre, Korea University, Tokyo, Japan; 9 Laboratory of Food and Environmental Hygiene, Veterinary Medicine, Gifu University, Gifu, Japan; 10 Laboratory for Conservation and Utilization of Bio-resource and Key Laboratory for Microbial Resources of the Ministry of Education, Yunnan University, Kunming, China; Universita degli Studi di Pavia, ITALY

## Abstract

Genetics of pigs has been well studied in Europe and Asia, but most of previous studies of molecular phylogeny of *Sus scrofa* have been based on sequences of both wild and domestic forms. In this study we analysed genetic traits of *Sus scrofa* from 13 regions in Asia (including previously undisclosed Eastern Caucasus and Trans-Baikal regions) using purely wild boar samples. Mitochondrial control region and Y-chromosome genes (AMELY & USP9Y) were employed to resolve phylogeographic relationships. We discussed spatio-temporal dynamics of wild boar distribution and compared molecular data to morphological and cytogenetic data on wild boar variability and taxonomy. A total of 51 haplotypes were detected in mtDNA control region and five haplotypes were found in combined sequences of Y-chromosome genes. The phylogeography of Asia-wide wild boars supported a hypothesis of migration from South-East Asia to South Asia, followed by migration to East and West Asia. We present a hypothesis about independent dispersal of wild boars into West Asia from South and North-East Asia. Mitochondrial DNA phylogeny generally fits the morphologically based intraspecies taxonomy. Distribution of chromosomal variants of wild boar presently does not show clear correlation with mtDNA clades.

## Introduction

Wild boar (*Sus scrofa*), the ancestor of the domestic pig, is one of the most widely distributed mammals. It is distributed throughout Eurasia from Europe to the Far East, including South and East Asia, and extending to North Africa. This species was also introduced into the Americas, Australia and Oceania [1]. Because of the relationship with human settlement and movement, studies on *Sus scrofa* phylogeography have provided important evidence revealing both anthropogenic and biogeographical history [2]. The taxonomy of the genus *Sus* is contradictory due to variability of species’ morphological and genetic traits.

According to Keuling et al. [3] 16 wild boar subspecies are recognized based on morphological parameters, such as shape of skull, size and proportions of body, hair colour. These subspecies are divided into European, Asian and South-Asian groups. The group which could be conveniently called European includes *S*. *s*. *scrofa* L. 1758, *S*. *s*. *meridionalis*, Forsyth Major, 1882 and *S*. *s*. *attila*, Thomas, 1912. Geographical range of these subspecies includes Europe together with islands, and in North Africa some researchers recognize *S*. *s*. *algeri*, Loche, 1867.

The Asian group (Northern and North-Eastern Asia) includes *S*. *s*. *lybicus* Gray, 1868 (Transcaucasia and Near East), *S*. *s*. *nigripes* Blanford, 1875 (Central Asia to Western Mongolia), *S*. *s*. *sibiricus* Staffe, 1922 (North Mongolia, Baikal and Trans-Baikalia to west of Big Khingan), *S*. *s*. *ussuricus* (sometimes also named *coreana*) Heude, 1888 (Russian Far East, North-Eastern China, Korean peninsula) and *S*. *s*. *moupinensis* Milne-Edwards, 1871 reported from Coastal China south to Vietnam and west to Sichuan, but it is possible there are actually several subspecies involved [3]. Wild boars, inhabiting Japanese islands and Taiwan (subspecies *S*. *s*. *leucomystax* Temminck, 1842, *S*. *s*. *riukiuanus* Kuroda, 1924 and *S*. *s*. *taivanus* Swinhoe, 1863, also called *S*. *s*. *jubatus*) are also often included in “Asian” group. The South-Asian group (India and Pakistan) includes *S*. *s*. *davidi* Groves, 1981 and *S*. *s*. *cristatus* Wagner, 1839. Finally, the subspecies *S*. *s*. *vittatus* inhabits Indonesia and Peninsular Malaysia.

Cytogenetic studies of European (*S*. *s*. *scrofa*, Belarus, Western Russia), Trans-Caucasian (supposedly, *S*. *s*. *attila*) and Asian (Kyrgyzstan, Tyva and Russian Far East) animals revealed the East-West gradient in the diploid number of chromosomes: while wild boars from Russian Far East, Trans-Baikal and Tyva regions had almost exclusively 2n = 38 (like domestic pigs), in European wild boars there is a high proportion of animals with 2n = 36, resulting from a centric fusion (Robertsonian translocation, Rb) of the 15th and 17th chromosomes [4–6]. In wild boars from Kyrgyzstan (Central Asia) both 2n = 36 and 2n = 38 variants were found, but, in contrast of European wild boars, the 2n = 36 in Central Asian wild boars resulted from a centric fusion (Rb) of 16th (but not 15th) and 17th chromosomes [4]. The third chromosomal variant with 2n = 37 found in wild boars from Europe and Kyrgyzstan resulted from partial reduction of the 17th pair of chromosomes. Thus, from cytogenetic point of view East Asian (east to Lake Baikal) wild boars are clearly distinct from Central Asian, West Asian and European animals, while the last regions are similar in total number of chromosomes but differ in types of chromosomal variations.

Immunogenic studies also revealed significant differences between different subspecies (and groups of subspecies) of wild boar. Particularly, studies of antibodies of 10 genetic markers of blood type revealed polymorphism, intraspecies differentiation and specific features of European, Caucasian, Central Asian and Far Eastern wild boars. Far Eastern wild boars differed significantly from European animals; Caucasian wild boars were very similar to those from Eastern and Central Europe, while samples from Central Asia occupied intermediate position between European and Far Eastern wild boars [7]. Thus modern intraspecies taxonomy based on geographical distribution, morphology, cytogenetic and immunogenic traits recognizes generally European, Central Asian and Far Eastern groups of wild boar subspecies and about 10–11 subspecies are described from Asia.

From the point of view of molecular phylogeny at a large geographical scale, wild boars are genetically divided into Asian and European clades [8–11], which have split during Mid-Pleistocene 1.6–0.8 Ma ago [12]. Wild boars from East and South-Eastern Asia generally have greater amounts of genetic variation than European wild boars, based on both mtDNA [9] and nuclear markers [13]. Island South-Eastern Asia (ISEA) and mainland South-Eastern Asia (MSEA), known to be the area of phylogenetic origin of wild boars (*S*. *scrofa*), is a biodiversity hotspot where most other species in the genus *Sus* are present [14]. Genome sequence analysis suggests that extensive gene flow occurred during the glacial period [15] between the *Sus* species. Wild boars from MSEA, specifically the so-called “Mekong region” in the study by Wu et al. [16], contained nearly all major East Asian lineages with high genetic diversity relative to those from other regions of Asia.

While genetics of Far Eastern wild boars has been studied [13, 16–18], data from Central Asia are scarce. Near Eastern and Trans-Caucasian wild boars were treated as being closer to the European group [13, 19], however recent study of wild boar in Iran [20] showed presence of animals with haplotypes belonging to “Asian” clade. Status of wild boar in Caucasus is unclear, though immunogenetics and cytological data suggest it being closer to European than to Asian wild boars [7] and this conclusion has been recently supported by molecular data [21]. Also there are no data on the DNA polymorphism of wild boars from Central Siberia, where animals are supposed to belong to the subspecies *S*. *s*. *sibirica*, neither there is information of the genetic traits of wild boar for the territory from Western Tyan-Shan’ to Caspian sea (subspecies *S*. *s*. *nigripes*).

It is important to note that most of the previous studies of *Sus scrofa* phylogeography focused on wild boars and domestic pigs demonstrating both effects of natural expansion, domestication and animals’ translocations. In this study, we aimed to investigate the phylogeography of the mtDNA and Y-chromosome genes based on purely wild boar samples. This could allow detecting presumably natural trends in the distribution of both maternal and paternal lineages and excluding possible intervention of domestic pigs in phylogeographic inference of wild boars. We included wild boars from the Eastern Caucasus and Trans-Baikal (regions that haven’t been presented in previous studies), extensive samples from Eastern Asia and literature data to better delineate the genetic relationships within and among geographical regions. On the basis of the geographically extensive sampling we aim to i) build a hypothesis about possible ways of species expansion in Asia and ii) compare the molecular data with intraspecies taxonomy based on morphological and cytogenetic traits.

## Materials and methods

### Sample collection and DNA extraction

A total of 193 wild boar (*S*. *scrofa*) samples were collected from 13 locations across nine countries in Asia and Eastern Europe (Russia, n = 32; Estonia, n = 6; Mongolia, n = 17; North Korea, n = 2; South Korea, n = 73; Japan, n = 14; China, n = 15; Vietnam, n = 13; and Indonesia, n = 21) (Table 1). Samples from the new study areas namely Trans-Baikal region (Mongolia, Chitinskaya oblast), East-Caucasus region (Dagestan) were included. The experimental work was conducted with permission by the Conservation Genome Resource Bank for Korean Wildlife (CGRB) in Seoul National University where the wild boar genetic samples for this study are deposited. The Seoul National University Institutional Animal Care and Use Committee (SNUIACUC) do not have specific guidelines for wildlife sample collection, but recommends following the related laws in experiments using wild animal samples. Wild boar is classified as game animal in all the countries involved in the sampling in this study, and all the wild boar samples are either donated by hunters with hunting license in each country or collected from carcasses of accident-killed animals. Since no animals were killed for the purpose of this study, we did not attempt to seek approval from ethical review boards in the participating countries. However, the procedures involving animal samples were in accordance with the legal system in each country. Genomic DNA was extracted using the DNeasy Blood & Tissue Kit or the Gentra Puregene Tissue Kit (QIAGEN, USA) according to the manufacturer’s instructions.

**Table 1:** Sample locations and haplotype distributions of wild boars (*S*. *scrofa*) from 13 regions of Eurasia.

Location (Abbr.)	mtDNA control region			Y-chromosome		
	N	h	Haplotypes	N	h	Haplotypes
**Russia** Primorsky (RUP)	18	5	WB39 (3), WB54 (1), WB69 (5), WB70 (4), WB88 (5)	9	1	Y_Hap02 (9)
**Russia** Chita (RUC)	4	2	WB76 (3), WB79 (1)	2	1	Y_Hap02 (2)
**Russia** Dagestan (DAG)	6	3	WB96 (3), WB98 (2), WB99 (1),	4	1	Y_Hap04 (4)
**Estonia** (EST)	6	2	WB97 (2), WB98 (4)	0	ㅡ	ㅡ
**Mongolia** (MON)	10	7	WB39 (3), WB43 (1), WB71(1), WB72 (1), WB73 (1), WB76 (2), WB77 (1)	12	2	Y_Hap02 (11), Y_Hap05 (1)
**North Korea** (NKR)	2	1	WB62 (2)	0	ㅡ	ㅡ
**South Korea** Mainland (SKR)	40	6	WB55 (1), WB56 (14), WB62 (11), WB65 (12), WB84 (1), WB95 (1)	25	1	Y_Hap02 (25)
**South Korea** Jeju Island (KJJ)	29	2	WB50 (28), WB62 (1)	7	1	Y_Hap02 (7)
**Japan** (JPN)	13	4	WB39 (7), WB40 (4), WB43 (1), WB80 (1)	4	1	Y_Hap04 (4)
**China** Xinjiang (CXJ)	7	2	WB83 (1), WB85 (6)	2	1	Y_Hap05 (2)
**China** Yunnan (CYN)	7	6	WB10 (2), WB75 (1), WB81 (1), WB85 (1), WB86 (1), WB87 (1)	5	2	Y_Hap02 (1), Y_Hap05 (4),
**Vietnam** (VIE)	13	3	WB82 (2), WB90 (6), WB94 (5)	4	1	Y_Hap02 (4)
**Indonesia**(IND)	16	8	WB91 (2), WB92 (1), WB93 (2), WB105 (1), WB106 (5), WB107 (1), WB108 (1), WB109 (3)	11	3	Y_Hap01 (1), Y_Hap02 (8), Y_Hap03 (2),
**Total**	171			85		

The haplotypes were obtained from the mitochondrial DNA control region and Y-chromosome. Here we present haplotypes basing of the sequences of 576-bp fragment since they were used for further phylogeographical analysis. Information about haplotypes of 1,014-bp fragment is presented in S1 Table. Two Y-chromosome genes (543-bp AMELY and 425-bp USP9Y) were combined for analysis. **N**—sample size; **h**—number of haplotypes. See S1 Table for sample details.

### PCR amplification and DNA sequencing

The primers pDF/pDR [21] were used to amplify the mtDNA control region (approximately 1,250-bp to 1,350-bp) (GenBank accession numbers KY 911550–911578, KY911581-911704, KY911707-911711, KY911713-911718, KY911730, KY911732-911737, KY911739-911743). A set of combined PCR for sex identification was performed before sequencing Y-chromosome genes. A portion of the *Sry* gene (SRYB) was amplified to identify males, and a region of *Zfy-Zfx* genes (P1-5EZ/ P2-3EZ) was amplified as a positive control to confirm the success of PCR [22, 23]. Two Y-chromosome genes–the 425-bp intron 24 of the ubiquitin-specific protease 9 (USP9Y) gene and a 543-bp region located in the amelogenin (AMELY) gene (GenBank accession numbers for AMELY KY911747-753, KY911761-911768, KY911771, KY911773-911842, KY911844; for USP9Y KY911850-911879, KY911881-911883, KY911885-911926, KY911928-911929, KY911931-911933, KY911935-911937, KY911939-911941, KY911948)–were amplified using the method of Ramírez et al. [13]. All polymerase chain reactions (PCRs) were carried out in a final reaction mixture of 30uL, containing 2.0mM MgCl_2_, 0.2mM dNTPs, 0.27uM of each primer, and 0.75U *i*-StarTaq^TM^ DNA Polymerase (iNtRON BIOTECHNOLOGY, S. Korea). For DNA sequencing, PCR products were further purified using the Zymoclean™ Gel DNA Recovery Kit (ZYMO RESEARCH, CA, USA) according to the manufacturer’s instructions. DNA sequencing was carried out using an ABI 3730XL DNA Analyzer (Applied Biosystems, Foster City, CA, USA). All the detailed information about the samples and haplotypes used in further analysis is presented in S1 Table.

### Data analyses

DNA sequences– 171 sequences for the mtDNA control region and 85 sequences for the Y-chromosome–were aligned using Geneious 5.3.6 software (Biomatters Ltd., http://www.geneious.com). A total of 1,014-bp fragment of control region mtDNA was obtained and used for estimation of genetic diversity. Phylogenetic reconstructions were based on a shorter fragment of 576-bp which allowed comparison of the data obtained in this study with published sequences. The mtDNA control region sequences (576-bp) from a total of 327 wild boars, including 156 published sequences from NCBI (see S1 Table), were aligned and used for phylogeographical analysis. We used the published data on Asian wild boars, including Near Eastern samples. We did not include in the analysis big volumes of published sequences of European wild boar since most haplotypes from Eastern and Western Europe were shown to belong to the same haplogroup E1 [24, 25] and could be represented by samples from Estonia (Eastern Europe).

The AMELY and USP9Y sequences from the Y-chromosome were combined for further analyses. The number of haplotypes (h), haplotype diversity (Hd) and nucleotide diversity (*π*) were computed using DnaSP 5.1 [26]. The Tajima D test [27, 28] and Fu’s Fs [29] were calculated from 1,000 simulated samples to demonstrate selective neutrality or population demographic expansion. Harpending's Raggedness index (*r*) [30] is based on the maximum number of mutational differences and frequencies of the allelic classes. It was obtained with 100 bootstrap replications under the model of sudden demographic expansion using Arlequin 3.1 software [31].

Phylogenetic trees were constructed according to the best-suggested model or algorithm as implemented in Mega 5.2 [32] and jModelTest 2.1.3 [33]. Both mtDNA control region and Y-chromosome data were analyzed by the same models. The Bayesian tree based upon posterior probabilities was constructed using the program MrBayes 3.2 [34]. The Hasegawa-Kishino-Yano (HKY) model and the General Reversible Time (GTR) model with gamma distributed invariant sites (G+I) were selected for construction of tree basing on 1,014-bp and 576-bp fragments respectively. Gaps were treated both as complete and partial deletions. Statistical bootstrap support for each node of all phylogenetic trees was based on 1,000 replicates. Markov chain Monte Carlo (MCMC) procedure was performed with one cold and three hot chains in two different runs. To obtain sufficient convergence of the log likelihood values (average standard deviation < 0.019 for mtDNA sequences and <0.008 for Y chromosome genes), the MCMC ran for 3 million generations for the mitochondrial control region and 1.2 million generations for the Y-chromosome, respectively. For each MCMC, a tree was sampled every 100 generations and the first 25% of each run was discarded as burn-in. The consensus trees were illustrated using FigTree 1.3.1 [35]. As outgroups, sequences of the warthog, *Phacochoerus aethiopicus*, (GenBank accession number: AB046876) and the bearded pig, *Sus barbatus*, (GenBank accession number: EU549796 & EU549794) were employed respectively for the mtDNA control region and Y-chromosome. Median-joining network for Y-chromosome haplotypes was constructed using package *pegas* in R [36].

The time of divergence (*T*) was estimated among clades of Asia-wide wild boars in Bayesian phylogenetic tree. The time of divergence was calculated using the equation, *T* = *K*/(2*r*), given by Li [37], where sequence divergence (*K*, substitutions/site) was derived through *p*-distance between groups with mean distance using Mega 5.2 [32], and *r* is the average mutation rate of the mtDNA control region (*r* = 12.6 ± 3.2) [38].

## Results

### Genetic variability of Asia-wide wild boars

A total of 51 haplotypes of the mtDNA control region were identified in 171 wild boars sampled across Asia and Eastern Europe (Table 1). Haplotype diversity as estimated by 1,014-bp fragment of control region of mtDNA (Hd) ranged from 0.069 in Jeju Island, South Korea (KJJ) to 0.956 in Mongolia (MON), and nucleotide diversity (*π*) ranged from 0.089% in KJI to 1.873% in Indonesia (IND). Although genetic diversity was variable among locations, it was generally high in wild boars from South-Eastern Asia. Tests for departure of neutrality (Tajima’s D, Fu’s Fs and Harpending’s raggedness index) demonstrated contradictory results that did not allow unambiguous conclusions about populations’ demographic history (Table 2). Negative values of Fu’s were shown for JPN, CYN and MON but only for MON the departure from neutrality was statistically significant (P<0.02). For another population from China CXJ the negative value of Tajima’s D was statistically significant (P<0.05). Similarly, Harpending’s r is low and statistically significant for SKR, VIE and IND (P<0.05). This allows to suggest recent demographic expansion in some parts of South-Eastern Asia like it was suggested by Hu et al. [39], but this could also be related to genetic hitchhiking [29].

**Table 2:** Genetic variability and demographic analyses for Eastern Europe and Asia-wide geographical regions (from north-west to south-east).

Location	mtDNA control region					Y-chromosome	
	Hd	*π* (%)	Tajima's D	Fu's Fs	r	Hd	*π* (%)
**EST**	0.533	0.106	1.032^NS^	1.723 ^NS^	0.787 ^NS^	-	-
**DAG**	0.733	1.044	2.091 ^NS^	5.310 ^NS^	0.276 ^NS^	0.000	0.000
**CXJ**	0.286	0.142	-1.486*	2.508 ^NS^	0.673 ^NS^	0.000	0.000
**RUC**	0.5	0.248	-0.797 ^NS^	2.598 ^NS^	0.750 ^NS^	0.000	0.000
**MON**	0.956	0.225	-0.356 ^NS^	-4.581*	0.073 ^NS^	0.167	0.052
**RUP**	0.81	0.402	1.426 ^NS^	2.742 ^NS^	0.149 ^NS^	0.000	0.000
**SKR**	0.728	0.555	1.871 ^NS^	5.912 ^NS^	0.217*	0.000	0.000
**KJJ**	0.069	0.089	-2.425*	3.009 ^NS^	0.876 ^NS^	0.000	0.000
**JPN**	0.731	0.17	-1.292 ^NS^	-0.431 ^NS^	0.097 ^NS^	0.000	0.000
**CYN**	0.952	0.463	-1.314 ^NS^	-1.269 ^NS^	0.125 ^NS^	0.400	0.124
**VIE**	0.782	0.59	1.322 ^NS^	4.677 ^NS^	0.308*	0.000	0.000
**IND**	0.883	1.873	1.787 ^NS^	3.748 ^NS^	0.092*	0.473	0.147

A total of five haplotypes were detected in combined sequences (968-bp) of two Y-chromosome genes (Table 1). Four nucleotide-variable sites (all transitional changes) were identified. Most geographic locations, except Mongolia (MON), Yunnan in China (CYN), and Indonesia (IND), exhibited a single Y-chromosome haplotype (Tables 1–3). Haplotype Y_Hap02 was the most common in *S*. *scrofa*, comprising over 60% of the total (Table 3).

**Table 3:** Frequencies of Y-chromosome haplotypes of *S*. *scrofa* from 13 geographical regions.

Location	Y_Hap 01	Y_Hap 02	Y_Hap 03	Y_Hap 04	Y_Hap 05	N
**DAG**	0.000	0.000	0.000	1.000	0.000	4
**CXJ**	0.000	0.000	0.000	0.000	1.000	2
**RUC**	0.000	1.000	0.000	0.000	0.000	2
**MON**	0.000	0.917	0.000	0.000	0.083	12
**RUP**	0.000	1.000	0.000	0.000	0.000	9
**SKR**	0.000	1.000	0.000	0.000	0.000	25
**KJJ**	0.000	1.000	0.000	0.000	0.000	7
**JPN**	0.000	0.000	0.000	1.000	0.000	4
**CYN**	0.000	0.200	0.000	0.000	0.800	5
**VIE**	0.000	1.000	0.000	0.000	0.000	4
**IND**	0.090	0.720	0.180	0.000	0.000	11
**Mean**	0.008	0.622	0.016	0.182	0.171	85

N, number of sequenced individuals; See Table 1 for location abbreviation.

### Phylogeography and divergence time of Asia-wide wild boars

In this study, 171 mitochondrial control region sequences were combined with 156 published wild boar sequences. A total of 51 haplotypes were detected in 171 sequences for 1,014-bp fragment and a total of 119 haplotype were detected in 327 sequences for 576-bp fragment (including five sequences with nucleotide ambiguity codes).

**Fig 1:**
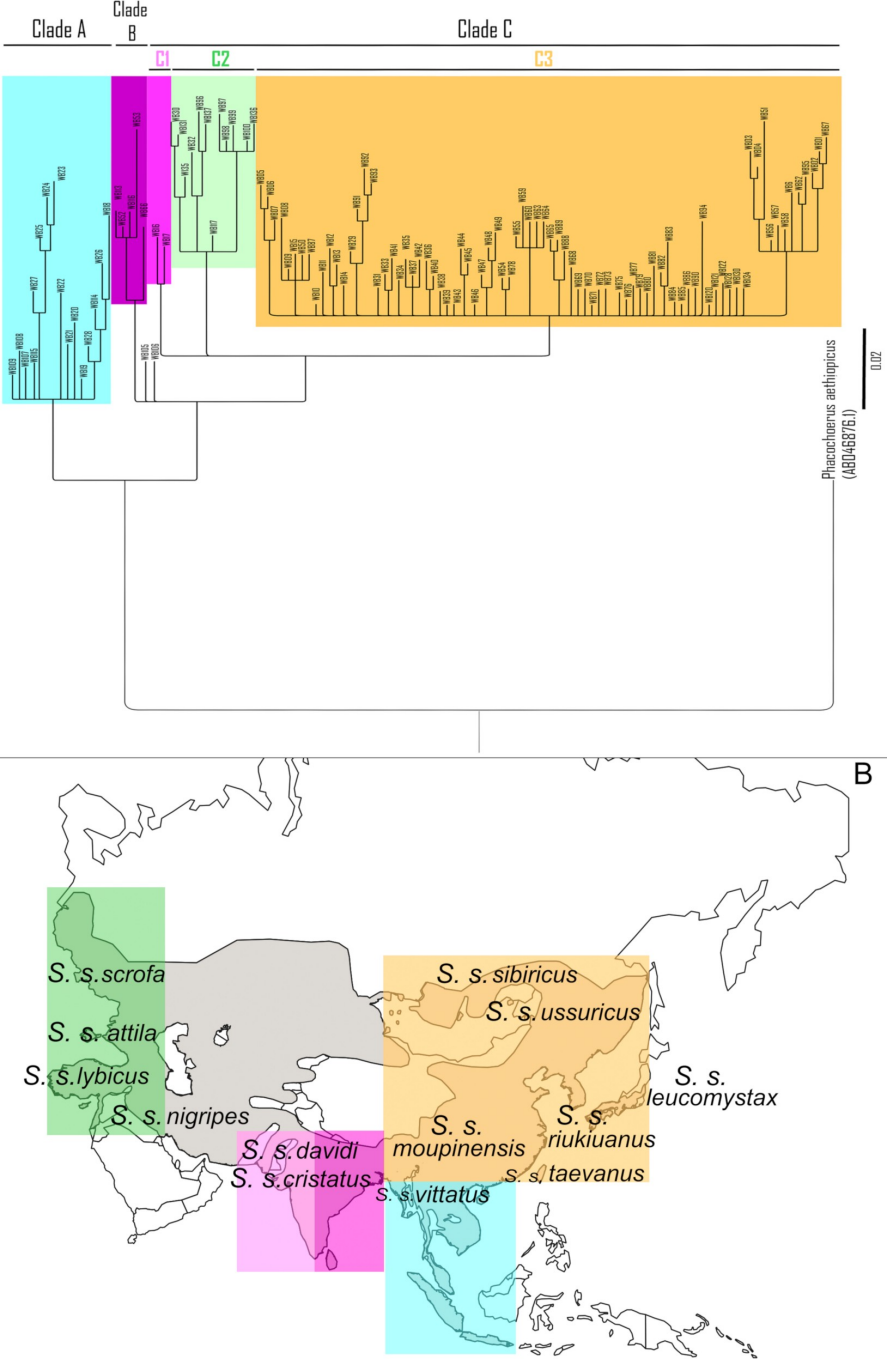
A. Bayesian (MCMC) haplotype tree based on the partial sequences of mtDNA control region (576-bp) of Asia-wide wild boar. The partial sequences of mtDNA control region (576-bp) obtained from GenBank and this study (n = 327) were employed for the tree reconstruction. General Time Reversible (GTR) + G + I model was implemented. Bayesian posterior probability is shown for branches with over 50% support. See S1 Table for haplotype information. B. Geographical distribution of wild boar subspecies and mitochondrial clades. The figure was created using wild boar range map available at https://commons.wikimedia.org/wiki/File:Sus_scrofa_range_map.jpg (public domain).

The Bayesian trees for both 1,014-bp fragment (S1 Fig) and 576-bp fragments (Fig 1A) both shows the ancestral position of some Indonesian haplotypes and the distinctness of Estonian and Dagestan haplotypes. The differences in topology between 1,014-bp and 576-bp trees are related to differences in datasets, particularly to inclusion of sequences from India and Western Asia (Iran, Turkey) in the dataset for 576-bp fragment. Since the data on the last fragment allows comparison of our data to those by other authors we further report and discuss in details the phylogeny based on it.

The whole set of haplotypes detected in 576-bp fragment split into three big clades. Clade A includes samples from ISEA. This clade was synonymous to the basal ISEA clade in Larson et al. [9]. It included mainly the haplotypes previously reported by Larson et al. [9] but also three haplotypes from our study (WB107-109). Clade B consists of samples from India, Nepal and Pakistan (North Hindustan peninsula) previously reported by Larson et al. [9] and thus it is synonymous to their “Hindustan” clade. Two Indonesian haplotypes (WB105 and WB107) first reported in this study were situated on the basal branch connecting Clade B to the clade A and their common ancestors. Clade C includes haplotypes from the rest of localities, representing continental Eurasia and Pacific islands (Japanese archipelago and Jeju Island).

Clade C in turn split into three clades. One of them, clade C1, includes samples from India, Nepal and Pakistan. This clade is geographically similar to clade D3 in Larson et al. [9], however in our tree it is clearly separated from the East-Asian and North-Asian samples. The position of sub-clade C1 haplotypes indicates its’ genetic proximity to clade B represented also by haplotypes found in Hindustan peninsula.

Clade C2 consists of haplotypes originating from Eastern Europe and Western Iran. It splits into 3 small sub-clades. One of them (haplotypes WB30, WB131, WB135) corresponds to clade NE1 in Khalilzadeh et al. [20]. The second one (WB32, WB96, WB137) corresponds to clade NE2 in the same study. It includes one haplotype from Dagestan (WB96) represented by 3 individuals. The third sub-clade consists of samples from Eastern Europe (Estonia), Dagestan and Iran. The Iranian haplotype in this clade (WB136) was treated as belonging to European clade [20], which is supposedly analogous to the clade D1 in Larson et al. [9]. Presence of Estonian haplotypes in this sub-clade supports this suggestion. Dagestan in this clade is presented by haplotypes WB97-98. One haplotype WB100 included in C2 geographically refers to South Korea and was taken from Cho et al. [21], but this was the “nucleotide ambiguity” sample (see S1 Table).

Clade C3 includes samples mainly from Eastern Asia (China, Trans-Baikal, Russian Far East, Korean peninsula, Indochina, Japanese islands) but also from Eastern Iran. It looks similar to clade D2 in Larson et al. [9] but including Iranian samples to this clade extends it to the west approximately to Caspian Sea. Thus it looks more like Asian clade in Khalilzadeh et al. [20]. Similar to the tree based on 1,014-bp fragment, no clear geographical structure could be detected within this clade. Most samples are rooted in basal branch; few compact clusters include haplotypes from different regions. The distal cluster within Clade C3 consists of samples from China, Japan and Indochina, but generally it received not very high (about 50%) support, thus we do not consider it as a separate clade.

**Fig 2:**
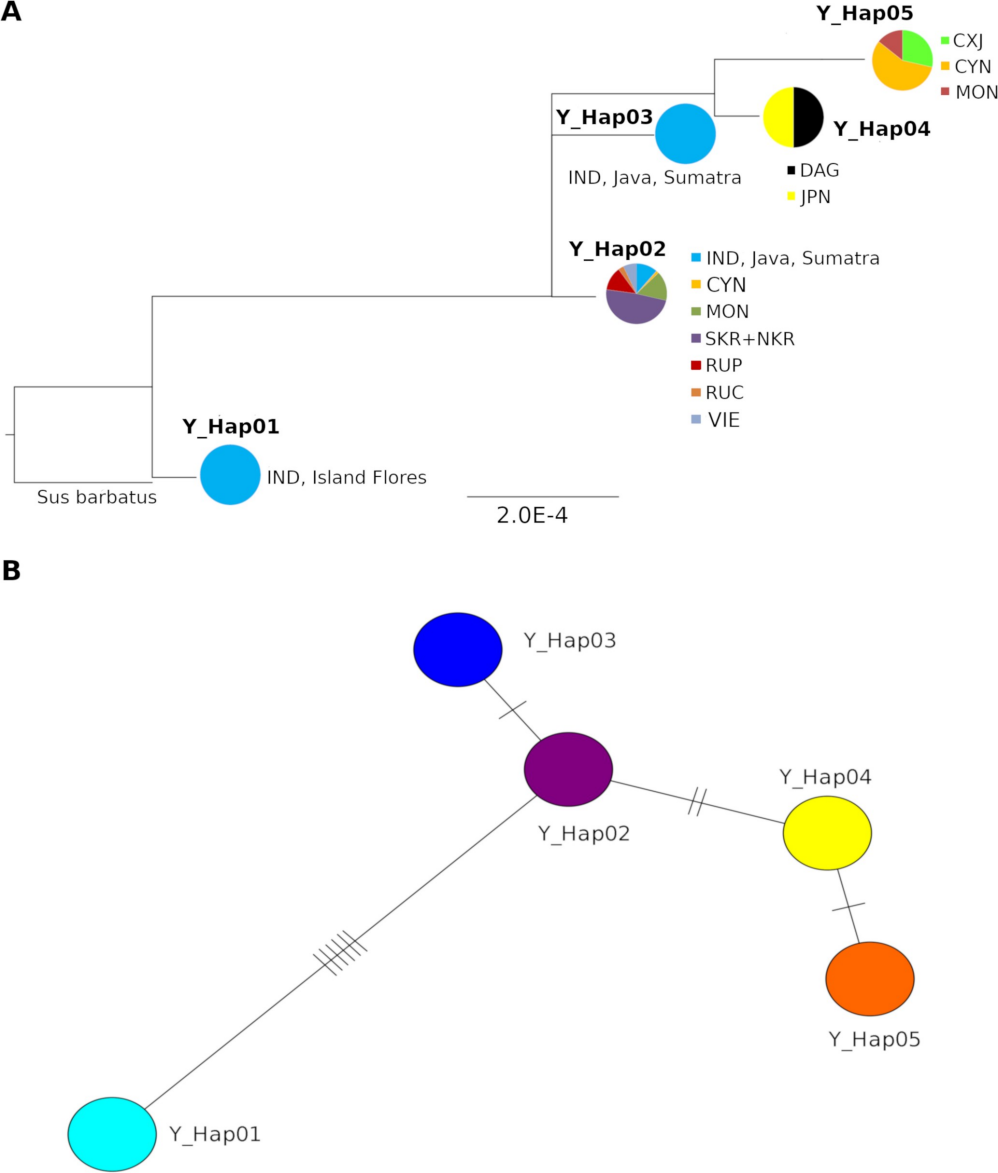
A. Phylogenetic tree based on combined sequences (968-bp) of two Y-chromosome genes (543-bp AMELY and 425-bp USP9Y) of Asia-wide wild boar. B. Median-joining tree of haplotypes of Y-chromosome genes (543-bp AMELY and 425-bp USP9Y) of Asia-wide wild boar. 85 Asia-wide wild boars (*S*. *scrofa*) were used. See Table 1 for location abbreviations.

In the Y-chromosome phylogenetic tree (Fig 2A) Indonesian *Sus scrofa* from Flores Island occupied basal position. The most common haplotype (Y_Hap02) was shared by wild boars from all regions of Asia, including ISEA (Table 3). Haplotypes Y-Hap03, Y-Hap04 and Y-Hap05 descended from Y_Hap02 (Fig 2B). The last two haplotypes were very similar differing by a single nucleotide substitution, but they differed significantly in geographical distribution. Haplotype Y_Hap05 was found in the samples from South-Western China while Y_Hap04 was the only one found in Japan on the islands Honshu and Kyushu and, interestingly, in Caucasian mountains (Dagestan, DAG), but not in the other regions of Asia. In differ with the mtDNA phylogeny the Y-chromosome tree shows that most of Chinese haplotypes are more similar to samples from Dagestan than to those from Eastern Asia. Besides, Dagestan haplotypes descend from Y_Hap02, while in mtDNA tree they are in the clade C2 that split from ancestral haplotype independently of East Asian group.

Sequence divergence time of Asia-wide wild boars was estimated based on clustering in the Bayesian phylogenetic tree (Fig 1A). The Clade B (India, Nepal, Pakistan) initially diverged from the ISEA clade in *T* = 1119 **×** 10^3^ YBP, while clades B and C split about 1,069 **×** 10^3^ YBP). The clade C2 of Eastern Europe and West Iran diverged from the other lineages in Clade C about 968 **×** 10^3^ YBP, thus soon after the split of Clades B and C, which is consistent with the finding of Groenen et al. [40] that reported similar time of split between European and Asian genetic groups (1.6–0.8 MYA).

## Discussion

### Mitochondrial haplogroups of wild boar in Asia basing on partial sequences of control region

Mitochondrial phylogeny of wild boar in Eurasia has been constructed in a number of publications, beginning from Giuffra et al. [8] and Larson et al. [9]. More recent studies focused on European *Sus scrofa* [11, 24, 25], others addressed East-Asian [16, 21] and West-Asian [19, 20, 41] pigs and wild boars. Here we compare the structure of our phylogenetic tree based on 576-bp fragment of control region to those presented by other authors.

Most of the existing trees (see citations above and Fig 1A) use the data from ISAE and India presented in Larson et al. [9], thus most of them show that ancestral haplotypes could be found in these two regions. There is an ambiguity concerning the relationships between these two groups: some authors [9, 21] show that Indonesian haplotypes are ancestral to Indian ones, while in the other phylogenies [41] these are two sister clades. Some of the Indonesian haplotypes sequenced in our study grouped with those presented in Larson et al. [9] supporting the basal position of the ISAE haplogroup. The status of samples from Hindustan is partly different from that presented in Larson et al. [9]—the clade D3 is not within the continental haplogroup but is a separate cluster C1 sister to European (C2) and Asian (C3) clades. We think that such position of C1 more adequately reflects the status of Indian wild boars taking into account the intraspecies taxonomy (see also discussion below and Fig 1B).

Another ambiguity in the mitochondrial phylogeny of Eurasian wild boar is the position of European haplotypes in relation to the Asian clade. While some studies [9, 20] put it inside the Eurasian cluster, most authors [16, 25, 42, 41] show Europe-Near Eastern and East-Asian haplogroups to be the sister clades. In our tree based on 1,014-bp fragment (S1 Fig) the West-Asian (including Dagestan) and East European samples are rooted inside the Pan-Eurasian cluster, however in the tree based on the 576-bp fragment it is a separate clade C2. Thus the position of European haplotypes in relation to Asian is ambiguous and depends upon the molecular marker used as well as the sequences included in the analysis. For example, the 1,014-bp fragment did not include the Indian haplotypes and part of Iranian samples [20] and it possibly affected the topology of the tree. Increasing the number of samples from Central Asia as well as increasing the standard length of the fragment could help explaining this ambiguity. Below we build the hypothesis about the species’ dispersal basing on the 576-bp fragment since it allows wider geographical coverage.

It is interesting to mention that samples from Dagestan (Eastern Caucasus) split into European and Near Eastern subclades within clade C2. Previous studies has reported genetic connection of Trans-Caucasian (Georgia, Armenia) and Iranian samples to both Near Eastern and European clades [41]. Here we report the same connections for the area situated north from the Caucasian mountain ridge. Khederzadeh et al [43] suggested the possibility of genetic connection of Dagestan wild boars to Italian rather than to Central of East European haplotypes. Our data indicate connection of Eastern Caucasus to NE clade. We found that there was also genetic connection between Caucasus and Central and Eastern Europe.

### Hypothesis about the routes of wild boar dispersal in Asia and Near East

In this study wide geographical sampling, particularly in previously non-reported parts of Northern Asia, allowed us to hypothesize about possible routes of wild boar expansion through Asia. Previously Larson et al. [9] wrote “initial dispersal from this area (ISEA) into the Indian subcontinent was followed by subsequent radiations into Eastern Asia and a final, progressive spread across Eurasia into Western Europe” without hypothesizing about the possible routes of expansion. Wu et al. [16] suggested possible ways of expansion of pigs and wild boars within East-Asian regions but did not discuss how they could be related to western haplogroups. In the articles focusing on the genetic status of Near Eastern wild boars [20, 43] animals from Turkey, Iran and Trans-Caucasian countries were described as belonging to European, Asian or Near Eastern haplogroups, but the history and relationships between these groups have not been discussed. Thus patterns of wild boar expansion in Asia remain unclear [12].

**Fig 3:**
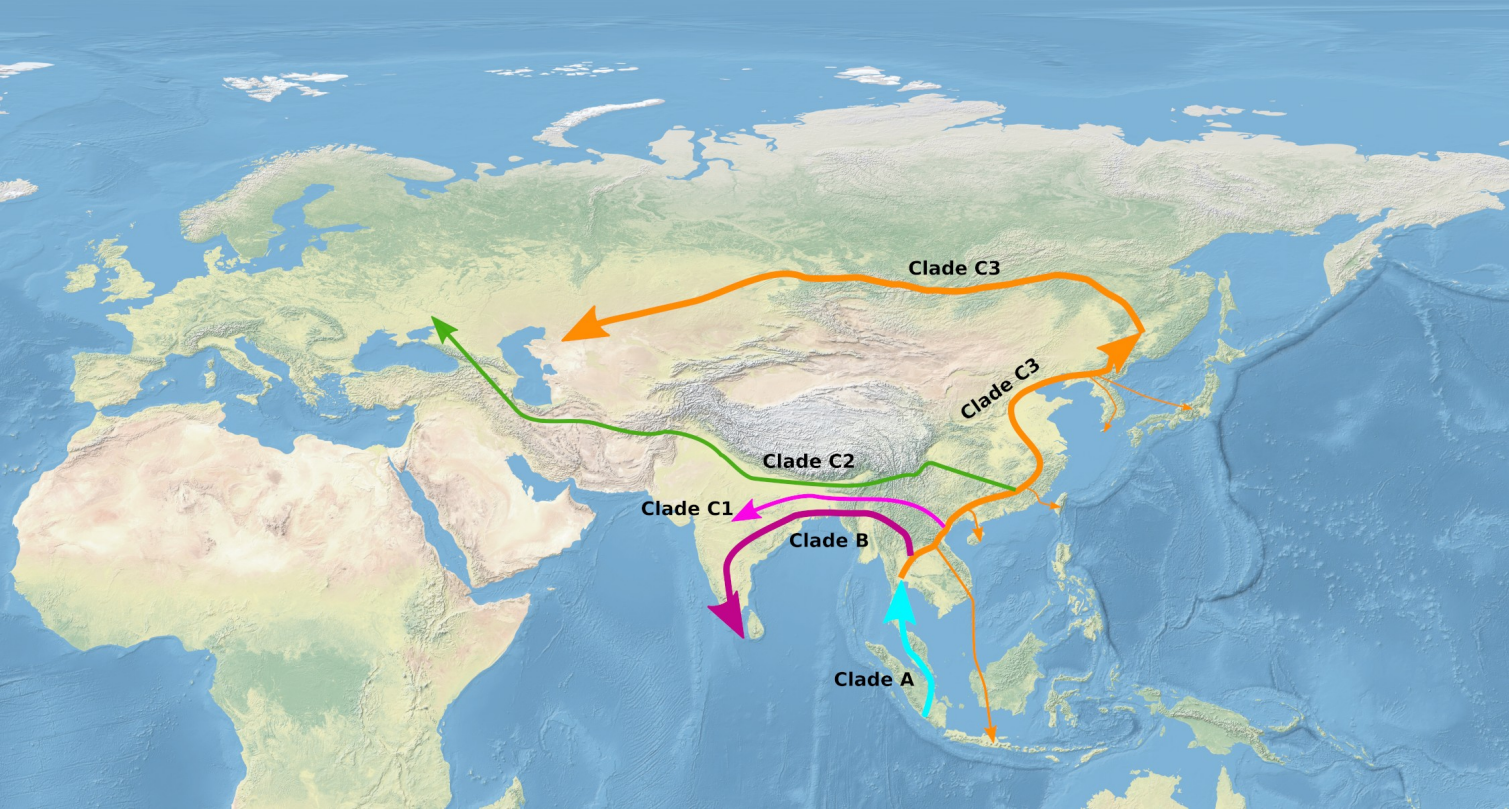
Suggested migration routes of wild boar in Eurasia. The map was made with Natural Earth. Free vector and raster map data @ naturalearthdata.com.

An analysis of both 1,014-bp and 576-bp fragment of the mtDNA control region supported the ancestral position of ISEA haplotypes to other Asian haplotypes previously reported by several authors [9, 16]. Suggesting the species *Sus scrofa* has an insular origin; animals could expand from the island of Sumatra to the mainland (Fig 3, Clade A). This suggestion is supported by the data of gene flow from Sumatra to Eastern Asia [15]. From Indochina animals could expand westwards to Hindustan resulting in the Indo-Nepal-Pakistan linages (Clades B and C1). These clades are the closest both to the ancestors of other Eurasian populations and to the ISEA clade.

The Cluster C2 which includes samples from Europe and Western Iran is a sister clade to the Asian cluster. This position of the European group is different from reported by Larson et al. [9] and Khalizadeh et al. [20] which put European group inside the Pan-Eurasian clade. On the other hand it agrees with the relative position of European and Asian cluster in Wu et al. [16]. This allows suggesting that West Asian lineage does not originate from East Asian lineage, but split independently from ancestral haplotypes and expanded west not through the Northern Asia, but in some other way. This lineage could expand to the south-west from Himalayas (Fig 3) to Asia Minor. Wu et al. [16] hypothesized that such split could occur in the upper stream of the Yangtze River in Western China. Groenen et al. [40] reported the existence of gene flow between Europe and North China which also favours the hypothesis of independent routes of dispersal: i) to Northern Asia and ii) to Near East and Europe. Guirao-Rico et al. [44] came to similar conclusions basing on modelling the migration patterns from the distribution of Y-chromosome haplotypes. They argued that the possible split between Asian and Eurasian haplotypes could take place in South-Western China. In this study we support the idea of independent dispersal of East-Asian and non-Asian (here Dagestan, Near East and European) haplotypes using the mtDNA data.

Our results do not agree with the scenario suggested by Cho et al. [21] that Korean wild boars originated from Kyushu Island, Japan by dispersal through land bridges to the Korean peninsula during the glacial periods in the Late Pleistocene. There are several clusters within clade C3 which include haplotypes from South Korea, which possibly means there were several waves and possible sources of wild boar expansion to Korean peninsula. The biggest and most distinct cluster is associated with haplotypes from South Asian (Vietnam, Myanmar, Burma), but not with Japanese samples. The last are also presented with several groups of haplotypes, which suggest that different islands of Japanese archipelago could be independently colonized from the mainland during cold periods when the sea level was low. Presence of Indonesian haplotypes within clade C3 indicates possible backward expansion or translocation of mainland pigs to ISEA.

### Geographical distribution of Y-chromosome haplotypes

Our analysis of phylogeography of Y-chromosomes is the first example of geographically extensive study based on purely wild boar samples without the inclusion of domestic pigs. In total the Y-chromosome haplotypes were sequenced for 85 individuals from Asia which is at least four times more than previously reported samples [13, 44].

The Y-chromosome phylogenetic tree in part supports the tree based on mtDNA–it clearly shows the ancestral position of the ISEA (Haplotypes 6 and 5). Similar to Ramirez et al. [13] haplotypes Y-Hap05 and Y-Hap04 were found presumably in West-Asian populations, while Y-Hap02 was found throughout Continental Asia. The fact that haplotype Y-Hap04 was the only haplotype found in Dagestan and Japan, and not found in other Asian population could be related to small regional sample sizes (n = 4 for each population). Similarity in haplotypes found in Caucasus and Japan could result from artificial translocations of animals, most probably from Europe and Near East to Japan. This fits to the results by Guirao-Rico et al. [44] about recent gene flow from non-Asian to Asian populations, particularly to Japan. However, if it is not an artefact resulting from small sample size or translocations, then these haplotypes could represent rare ancient haplotypes distributed in Southern Asia, which preserved in Western Asia and in island population in Japan. The genetic similarity between westernmost populations and animals from Pacific island has been described previously for Siberian roe deer [45]. If it is the case for Asian wild boar this would be another argument in support for the hypothesis of early split of lineage which gave origin to modern Near East and European haplotypes. Extensive sampling is needed to support or reject this hypothesis.

### Comparison of molecular, cytogenetic and morphological diversification in Eurasian wild boar

Larson et al. [9] mentioned that East-West split of mtDNA lineages is consistent with morphologically based studies, which have highlighted the distinctiveness of animals from South-Eastern Asia (*Sus scrofa vittatus* in particular). This statement, to our best knowledge, was the only one concerning the correlation between molecular and morphological phylogeny until Keuling et al. [3] addressed it in their review of the biology and systematics of Eurasian wild boar. Basing on previously published mtDNA phylogenies they suggested elevation of several subspecies of wild boar to the status of species. Here we discuss this problem basing on bigger samples of pure wild boar and large geographical scale of our study.

According to our results the phylogenetic tree based on the sequences of control region of mtDNA only partly agrees with the intraspecies taxonomy, based on morphological traits (Fig 1B). Particularly, the group of Indian, Nepal and Pakistan haplotypes covers the ranges of two subspecies *S*. *s*. *davidi* and *S*. *s*. *cristatus* which agree with presence of Hindustan haplotypes in two clades–Clade B and Clade C1. Subspecies *S*. *s*. *ussiricus* and *S*. *s*. *sibirica* could not be well distinguished basing on our data, as well as other subspecies from Eastern Asia. Most of the samples from their range are in Clade C3 together with samples from North-Eastern Iran where the subspecies *S*. *s*. *lybicus* was described. Distal South-Asian cluster within Eurasian clade could be suggested to represent the subspecies *S*. *s*. *moupinensis* reported from Coastal China south to Vietnam and west to Sichuan, but this cluster has received low support. Animals from Europe and Caucasus belonging to the subspecies *S*. *s*. *scrofa* and *S*. *s*. *attila* fall into the Clade C3. Within it the cluster including both East-European and Iranian samples could represent *S*. *s*. *scrofa*. Two other clusters could represent other subspecies found in Caucasus and Near East, like *S*. *s*. *attila* and *S*. *s*.*nigripes*, but more intensive sampling and additional analysis is needed to check this suggestion. Generally the mtDNA classification revealed less intraspecies groups than morphologically based classification, however supports the existence of European, Near Eastern, and Far Eastern groups of subspecies. The tree of Y-chromosome haplotypes in fact showed no divergence within the continental samples. This could result from relatively small number of samples from some regions, but also it could indicate that morphological and molecular differences in wild boar could evolve mainly due to female philopatry—a tendency to stay in or habitually return to a particular area [46, 47].

Cytogenetic classification based on chromosomal forms supports diversification of Western and Eastern clades, showing prevalence of 2n = 38 in the eastern and 2n = 36 in the western part of Eurasia. On the other hand several studies have shown that it is hardly possible to find a direct correlation between mitochondrial DNA diversity in wild boar and its cytogenetic variability. Particularly, Fang et al. [6] have shown that close genetic relationship between mtDNA haplotypes from wild boars with 2n = 36 to those from domestic pigs with 2n = 38. Arslan and Albayrak [48] reported 2n = 38 in wild boar from the central part of Turkey, where only Near Eastern and European haplotypes have been reported [49].

## Conclusions

This phylogeographic study of Asia-wide wild boars provided important insights into the evolutionary history and migration patterns of *S*. *scrofa* throughout Asia. Inclusion of wild boars from Caucasus and Trans-Baikal regions provided more comprehensive phylogeography of wild boars in Asia. Our data allow hypothesizing about the following routes of wild boar dispersal from mainland South-Eastern Asia:

- to the west to India, Nepal, Pakistan and Sri-Lanka;
- to the north, including China, Korea and Russian Far East and then westward up to Eastern Iran;
- to the west possibly through South-Western China, south of Himalayas to Near East and Europe, giving origin to European group of genetic lineages.

Paternal phylogeny supported the ancestral position of ISEA region, but did not show clear phylogeographic structuring between mainland lineages which could imply that male wild boars dispersed more actively than females, thus possibly impeding lineage sorting, but also could result from the small number of samples in the analysis.

Mitochondrial DNA phylogeny generally fits the morphologically based intraspecies taxonomy. At the same time some “morphological” subspecies could not be distinguished based on the modern mtDNA phylogenetic tree. This makes doubtful elevating the existing subspecies to species level as suggested by Groves [50] and supported by Keuling et al. [3]. Distribution of chromosomal variants of wild boar presently does not show clear correlation with mtDNA clades.

## Supporting information

S1 Fig Bayesian (MCMC) haplotype tree based on the partial sequences of mtDNA control region (1014-bp) of Asia-wide wild boar. 171 wild boars (Sus scrofa) sampled Asia-wide were used. The Hasegawa-Kishino-Yano (HKY) model with gamma-distributed invariant sites (G+I) was implemented. Highlighted samples indicate compact clusters and the geographical codes indicate regions included in the highlighted clusters. Non-highlighted samples are from different regions of North-Eastern Eurasia.
(TIF)

S1 Table List of mtDNA control region and two Y-chromosome genes (AMELY, USP9Y) sequences used for phylogenetic and phylogeographical analyses. Published 112 mtDNA control region sequences were obtained from GenBank.
(XLSX)
