# Supplementary figures and images for: Asia-wide phylogeography of wild boar (Sus scrofa) based on mitochondrial DNA and Y-chromosome: Revising the migration routes of wild boar in Asia
Source: PLoS One. 2020 Aug 24;15(8):e0238049. doi: 10.1371/journal.pone.0238049 (PMC7444817; doi:10.1371/journal.pone.0238049)

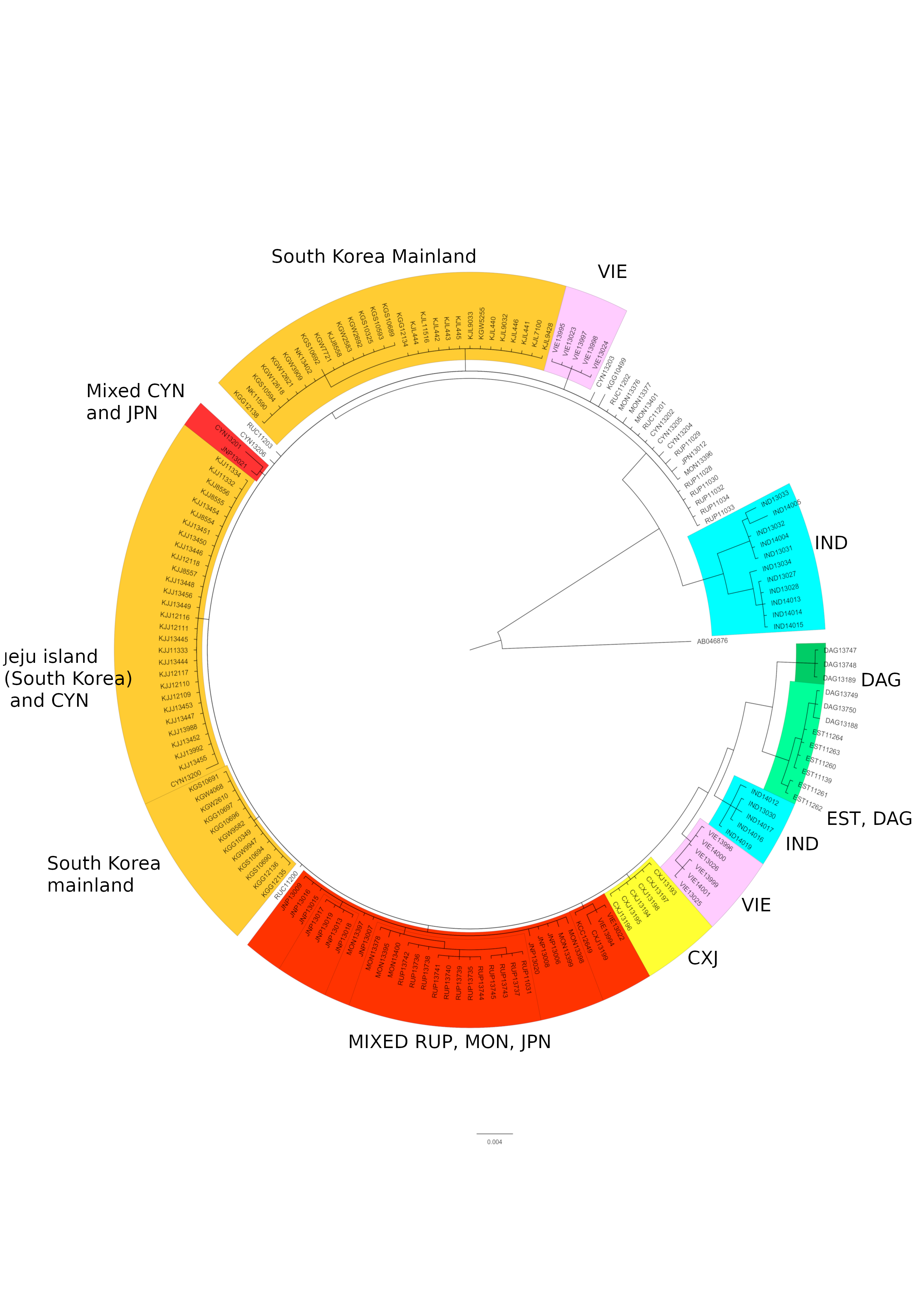

Supplement: S1 Fig — 171 wild boars (Sus scrofa) sampled Asia-wide were used. The Hasegawa-Kishino-Yano (HKY) model with gamma-distributed invariant sites (G+I) was implemented. Highlighted samples indicate compact clusters and the geographical codes indicate regions included in the highlighted clusters. Non-highlighted samples are from different regions of North-Eastern Eurasia. (TIF) [file pone.0238049.s001.tif]
